# Thermal, Morphological and Mechanical Properties of Multifunctional Composites Based on Biodegradable Polymers/Bentonite Clay: A Review

**DOI:** 10.3390/polym15163443

**Published:** 2023-08-17

**Authors:** António Benjamim Mapossa, Afonso Henrique da Silva Júnior, Carlos Rafael Silva de Oliveira, Washington Mhike

**Affiliations:** 1 Department of Chemical Engineering, University of Pretoria, Lynnwood Road, Pretoria 0002, South Africa; 2 Department of Chemical Engineering and Food Engineering, Federal University of Santa Catarina, Florianópolis 88037-000, SC, Brazil; 3 Department of Textile Engineering, Federal University of Santa Catarina, Blumenau 89036-002, SC, Brazil; 4 Polymer Technology Division, Department of Chemical, Metallurgical and Materials Engineering, Tshwane University of Technology, Pretoria 0183, South Africa

**Keywords:** biodegradable polymer, bentonite clay, thermal, morphological, mechanical properties

## Abstract

The extensive use of non-biodegradable plastic products has resulted in significant environmental problems caused by their accumulation in landfills and their proliferation into water bodies. Biodegradable polymers offer a potential solution to mitigate these issues through the utilization of renewable resources which are abundantly available and biodegradable, making them environmentally friendly. However, biodegradable polymers face challenges such as relatively low mechanical strength and thermal resistance, relatively inferior gas barrier properties, low processability, and economic viability. To overcome these limitations, researchers are investigating the incorporation of nanofillers, specifically bentonite clay, into biodegradable polymeric matrices. Bentonite clay is an aluminum phyllosilicate with interesting properties such as a high cation exchange capacity, a large surface area, and environmental compatibility. However, achieving complete dispersion of nanoclays in polymeric matrices remains a challenge due to these materials’ hydrophilic and hydrophobic nature. Several methods are employed to prepare polymer–clay nanocomposites, including solution casting, melt extrusion, spraying, inkjet printing, and electrospinning. Biodegradable polymeric nanocomposites are versatile and promising in various industrial applications such as electromagnetic shielding, energy storage, electronics, and flexible electronics. Additionally, combining bentonite clay with other fillers such as graphene can significantly reduce production costs compared to the exclusive use of carbon nanotubes or metallic fillers in the matrix. This work reviews the development of bentonite clay-based composites with biodegradable polymers for multifunctional applications. The composition, structure, preparation methods, and characterization techniques of these nanocomposites are discussed, along with the challenges and future directions in this field.

## 1. Introduction

In recent years, the use of products based on non-biodegradable, synthetic plastics has increased enormously. It has generated a high quantity of plastic waste which accumulates in landfills, causing significant environmental problems [1,2]. Plastic waste also finds its way into water bodies, disintegrating into microplastics which then manifest as microplastics in drinking water. Plastic waste is also consumed by marine life, resulting in adverse effects. Studies have shown that approximately 300 million tons of plastics derived from fossil hydrocarbon resources are produced annually worldwide, and this is expected to keep increasing [1,3].

**Figure 1:**
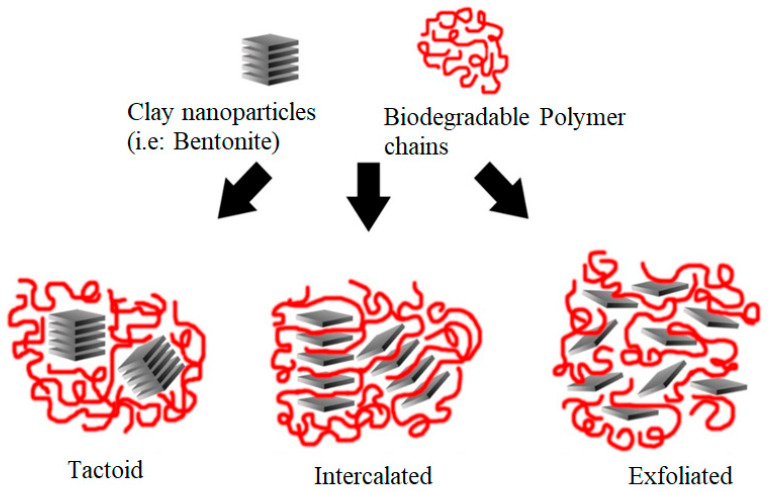
Schematic illustration of the nanocomposite structures. Three main types of morphology, including agglomerates, intercalated, and exfoliated structures of polymer-based clay nanocomposites can be obtained. Reprinted with permission from Ref. [30]. Copyright 2021, MDPI.

Due to the high degradation resistance and extended time for the decomposition of non-biodegradable polymers, these polymers have significantly contributed to environmental problems. To alleviate these problems caused by non-biodegradable polymers, the use of biopolymer and biodegradable polymers is a viable alternative due to the renewability of their sources, availability, biodegradability, and eco-friendliness [4,5,6]. Biodegradable polymers have similar properties to conventional polymers; thus, these can replace non-biodegradable conventional polymers in many applications such as packaging, medical devices, drug capsules, personal hygiene products, and agricultural applications [2]. Biodegradable polymers are largely derived from renewable agricultural feedstock such as starch and cellulose [7]. Although biopolymers are fully biodegradable, they present shortcomings such as relatively low mechanical resistance, inferior gas barrier properties, low processability, and poor economic viability related to their production. Thus, these properties must be improved to cover a vast range of properties provided by non-biodegradable polymeric materials [8]. This can be achieved by modifying biodegradable polymers through the incorporation of functional nanofillers such as clays into their matrices to form polymer nanocomposites. The incorporation of nanofillers aims to improve polymer properties such as thermal, mechanical, and other physical properties when compared to the virgin polymer [9,10,11,12,13] and consequently, to extend their applications in belligerent environments [13,14,15,16]. To accomplish the desired properties of polymer nanocomposites, the surface of inorganic nanofillers is typically modified to promote their uniform dispersion throughout the polymer matrix and enhance interfacial interaction with the organic matrix (Figure 1). Several works have reported the effect of sheet silicate clays (phyllosilicates) in modifying polymer matrices. Phyllosilicate montmorillonite has been studied widely as a functional nanofiller for polymer matrices [13,17,18,19]. This work reports on the recent advances in bentonite (a montmorillonite clay) as nanofillers for biodegradable polymer matrices. Bentonite is an absorbent aluminum phyllosilicate principally consisting of montmorillonite (MMT). It is a sedimentary rock comprising a high clay content with a typical 2:1 layered structure known as smectites containing sodium and calcium ions between the layers [20]. Montmorillonite, which belongs to the smectites group, is considered a major mineral for bentonite [20].

Bentonite also presents common impurities such as calcite, feldspar, quartz, mica, and organic matter. These offer a secondary impact on the thermal stability and cation exchange capacity of bentonites [20,21]. Among the traditional ceramics, bentonite is one of the inorganic nanofillers most used by researchers in different applications such as civil engineering, iron ore palletization, animal and poultry feed pelletization, paints, cosmetics, pharmaceuticals, and wastewater treatment [22]. This is justified because bentonite has a high cation exchange capacity and surface area [23], and other important properties such as thixotropy, hydration, swelling capacity, the capacity to bond, impermeability, and plasticity [22]. Bentonite is environment-friendly and available on a large scale. Is also relatively inexpensive. However, one of the principal concerns when incorporating nanoclays (e.g., bentonite) into a polymer matrix is the difficulty in uniformly dispersing them in the matrix. An entirely exfoliated structure (i.e., structure in which the silicate layers are fully and regularly distributed in the continuous polymeric matrix) is required to improve the mechanical properties. However, the tendency of silicate-based nanofillers to agglomerate within polymer matrices is generally difficult to overcome [24]. This is because most polymers are hydrophobic, whereas silicates are hydrophilic. Therefore, it is necessary to pretreat the clay prior to dispersing it in the polymer matrix to enhance its compatibility with the hydrophilic polymer [24,25,26]. The conversion of hydrophilic silicates into organophilic is one of the most reliable methods used to enhance the compatibility of clays and polymers. In this method, hydrated cations in the galleries of clays are substituted by cations from surfactants, e.g., alkyl or hydroxyl ammonium cations [27,28]. When this occurs, the basal spacing of the clay layers increases and the surface energy of the clays decreases. Thus, compatibility with hydrophobic polymers is improved and polymer chains can enter the galleries under defined processing conditions [24,29].

Junior et al. [2] reported in their study that the preparation of bionanocomposites with a small amount of inorganic filler was an effective procedure for enhancing some of the biodegradable polymer properties, such as thermal, mechanical, and barrier properties when compared to the corresponding neat polymer. Thus, bentonite is one of lamellar silicates mostly used by researchers as an inorganic filler due to the aforementioned reasons [2]. For the preparation of polymer–clay nanocomposites, different methods, such as solution casting, melt extrusion, in situ polymerization, are used. However, the choice of these methods depends on the type of nanofillers, interfacial interaction, and the level of dispersion of the nanofillers into the polymer matrix to obtain the desired biodegradable polymer nanocomposites for multifunctional applications.

The development of more efficient and cost-effective functional materials for industrial applications with a reduced environmental footprint, which are easier to process and are biodegradable at the end of their useful lives, has attracted more attention in academia and industry. Depending on the final properties, multifunctional biodegradable polymer nanocomposites can be suitable for various industrial applications such as electromagnetic (EM) shielding, energy storage, electronics, and embedded capacitor applications. This clay can be combined with other fillers (e.g., graphene) and incorporated into fully biodegradable polymers (e.g., polysaccharides) to develop exciting materials for flexible electronics applications. Therefore, this combination of bentonite clay with other fillers could significantly lower production costs when compared with only the use of carbon nanotubes or metallic fillers.

This work deals with the progress made in developing biodegradable polymer-based bentonite clay nanocomposites for multifunctional applications. Furthermore, a brief description of the composition and structure of bentonite clay, different preparation methods, and characterization of the thermal, mechanical, microstructure properties of biodegradable polymer-based bentonite nanocomposites are discussed. Finally, conclusions are drawn and new challenges are discussed.

## 2. Bentonite Clay: Classification, Composition, and Structure

**Figure 2:**
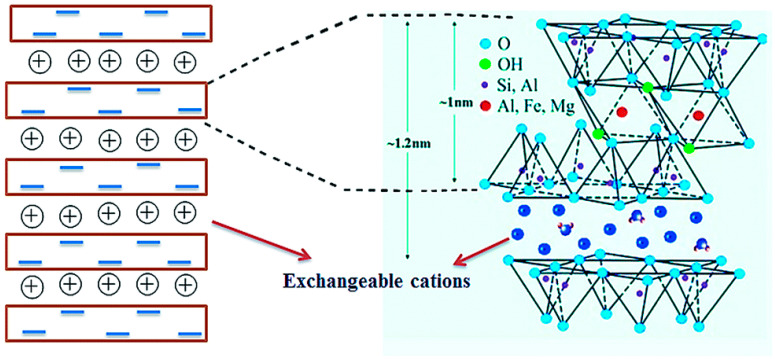
General structure schematic of bentonite clay. Reprinted with permission from Ref. [31]. Copyright 2014, Royal Society of Chemistry.

The structure of bentonite consists of two basic building blocks: the aluminum octahedral sheets and silica tetrahedral sheets, as illustrated in Figure 2. A single unit cell comprises one aluminum octahedral sheet sandwiched between two tetrahedral silica sheets [31]. The layered silicates present a slight negative charge, which is compensated by exchangeable cations in the intermediate layers. In addition, the charge is weak where cations Na^+^, Ca^2+^, and Mg^2+^ may be adsorbed with a connected hydration shell or hydration sphere. Thus, the cations contained in the clay can be substituted through ion exchange [31].

Generally, depending on the dominant exchangeable cations, there are two types of bentonite clays: sodium (Na) bentonite and calcium (Ca) bentonite types. Sanavada et al. [22] reported that in natural bentonite clay, if the content of ionic sodium (Na^+^) is more than 20%, that type of bentonite is called sodium bentonite. Moreover, if the concentration of ionic calcium (Ca^2+^) is more than 20%, that type is named calcium bentonite. Therefore, although both bentonite clays are swelling materials, the high tendency of water absorption from Na^+^ ions in sodium bentonite makes it prone to absorbing relatively large amounts of water, resulting in high swelling bentonite. In contrast, calcium bentonite, which contains Ca^2+^ ions, is less susceptible to water absorption, and is known as a low-swelling bentonite [32,33]. This is also supported by a study conducted by Muhammad and Siddiqua [32] that reported the cation exchange capacity (CEC) of the calcium bentonite with the value of 50 meq/100 g, which is lower than the high swelling-type bentonite (Na), with a range of 80–85 meq/100 g. Other differences include the surface area of the Ca bentonite clay, which is between 60 and 120 m^2^/g, while Na bentonite clay is about 20 to 30 m^2^/g [22]. The literature also reported the difference in concentrations of chemical compounds present in the calcium and sodium bentonites. The chemical compositions of bentonites were carried out using XRF measurements. The XRF data showed that the Ca bentonite clay is composed of CaO (4.88%), MgO (2.65%), Fe_2_O_3_ (6.35%), K_2_O (0.69%), Na_2_O (0.74%), TiO_2_ (0.64%), Al_2_O_3_ (16.44%), SiO_2_ (60.82%), and LOI (6.79%) [34], while the Na bentonite clay is composed of CaO (1.44%), MgO (2.75%), Fe_2_O_3_ (11.92%), K_2_O (0.29%), Na_2_O (3.22%), Al_2_O_3_ (15.34%), SiO_2_ (52.55%), TiO_2_ (1.62%), and LOI (9.80%) [35].

**Figure 3:**
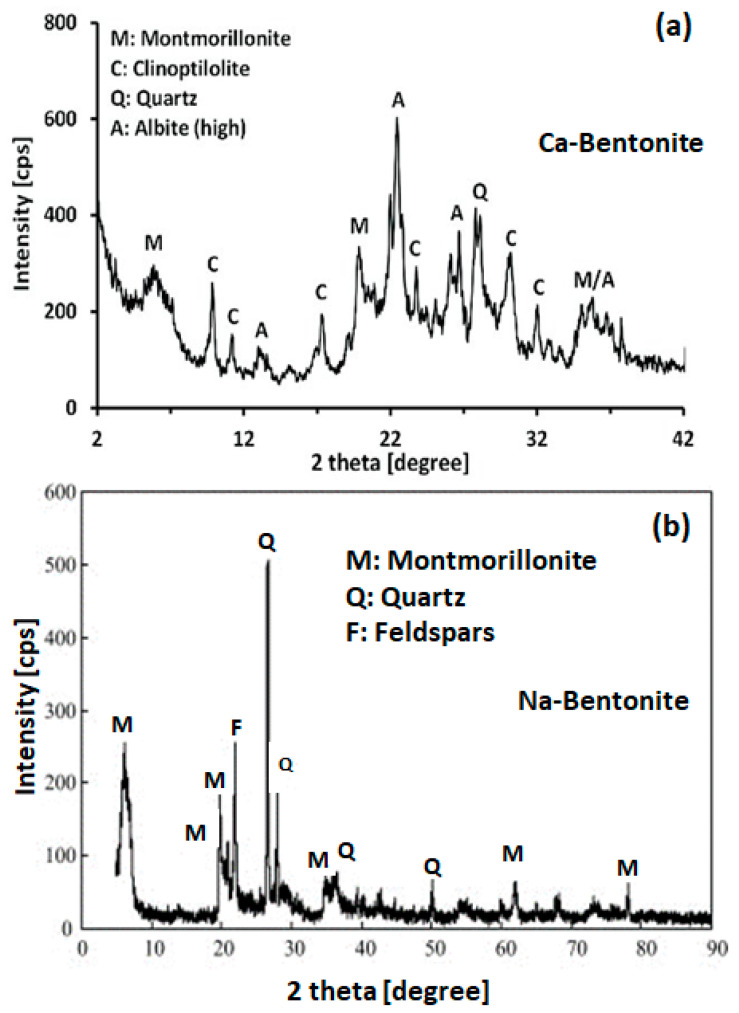
XRD pattern of: (**a**) Ca-bentonite clay. Reprinted with permission from Ref. [36]. Copyright 2020, MDPI; (**b**) Na-bentonite clay. Reprinted with permission from Ref. [37]. Copyright 2011, Elsevier.

The structural properties of Ca bentonite and Na bentonite were investigated using XRD. Through XRD studies, Choo et al. [36] showed that the principal clay mineral in Ca bentonite was montmorillonite (MMT). Other minerals such as quartz, albite, and clinoptilolite were identified (Figure 3a). In addition, using the XRD pattern of natural Na bentonite (Figure 3b), Zhirong et al. [37] demonstrated that the Na bentonite clay is also composed primarily of montmorillonite (MMT), with the characteristic features at *d*_001_ = 14.29 A° and *d*_020_ = 4.49 A°. The predominance of sodium is located on the basal spacing (*d*_001_ = 14.29 A°), which allows for the description of the samples principally as sodium bentonite (Na bentonite). Other peaks showing the occurrence of impurities such as quartz and feldspar were also observed in the XRD pattern.

## 3. Thermal Stability of Bentonite Clay

As previously reported, bentonite clay is obtained in various countries where it occurs superimposed on other mineral deposits. Therefore, the modification of bentonite has been performed using different organic compounds. This process is an important first step for preparing clay mineral based-polymer blends. It modifies the clay surface chemistry, altering its nature from hydrophilic to organophilic. It makes bentonite clay more compatible with organic polymers and allows for the exfoliation of the clay layers into the polymer matrix [38,39]. According to Massinga Jr et al. [39], quaternary alkylammonium salts that include bromides or chlorides are most intercalated in MMTs. Quaternary ammonium ions with long chains are ideal, as they lead to a larger interlayer spacing, allowing for facile penetration of polymer chains into the clay interlayer space. The co-intercalation of polymer chains assists the dispersion of distinct particles of bentonite clay through the polymer. Researchers have evaluated the thermal stability of modified bentonite clay to determine the exchanged content of organo-surfactant using thermogravimetric analysis (TGA) [38,40].

**Figure 4:**
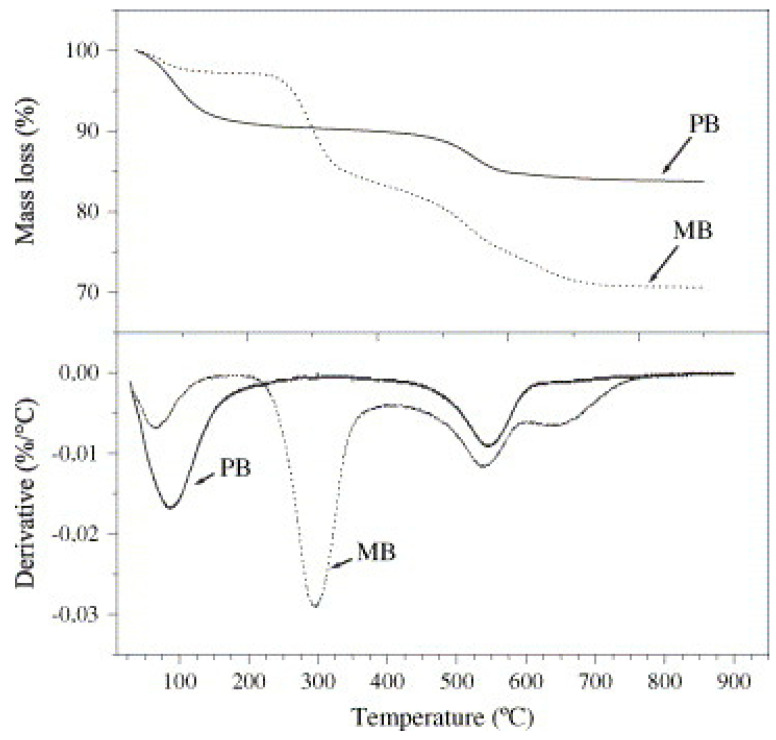
TGA and DTG curves of the unmodified bentonite (PB) and modified bentonite (MB). Reprinted with permission from Ref. [41]. Copyright 2005, Elsevier.

Ramos Filho et al. [41] compared the stability of unmodified Brazilian bentonite with its modified form (Figure 4). The authors reported that the first TGA mass loss of unmodified bentonite started at 30 °C and was completed at 200 °C. Therefore, the mass loss was attributed to water content via volatilization. In comparison, the second TGA mass loss of unmodified bentonite was observed between 430 and 634 °C due to the decomposition and dehydroxylation of the aluminosilicate. In addition, two mass losses were found for modified bentonite, which was attributed to organic salt decomposition. First, there was a TGA mass loss from 170 °C to 400 °C, and second, a TGA mass loss started at 575 °C and was completed at 800 °C. The authors also reported that the decomposition of organic salts occurs when the molecules of salt are located outside or within the interlamellar spaces and below the decomposition temperature of the neat quaternary salt. Furthermore, the unmodified bentonite (PB) water content was much higher than that for the modified bentonite (MB). This behavior was associated with the reduced hydrophilic nature of the clay after modification. For the modified clay, the organic salt amount was approximately 21%. Therefore, the value confirmed the efficacy of the modification procedure.

**Figure 5:**
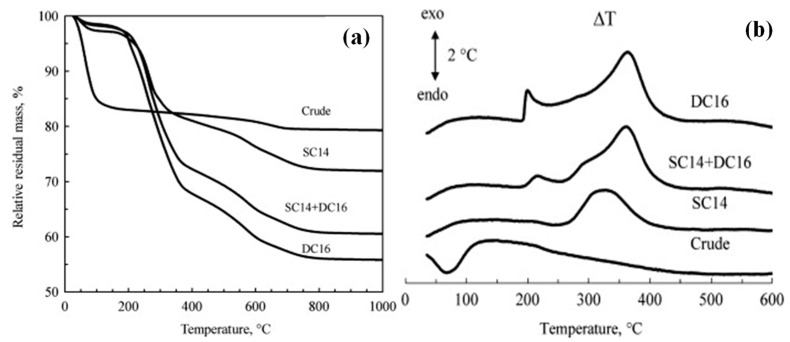
(**a**) TGA curves of untreated and organically treated bentonites. (**b**) DTA curves of crude and organically treated bentonites. Reprinted with permission from Ref. [39]. Copyright 2010, Elsevier.

Another study by Massinga Jr et al. [39] evaluated and compared the thermal stability of crude Mozambican bentonite with its modified form. The authors demonstrated that the TGA mass loss ensued a stepwise process in all samples and tended to increase with increasing organic salt content. Only two principal mass loss events were observed in the TGA of crude bentonite. In contrast, the TGA of the organo-bentonites demonstrated further thermal events (Figure 5a). The inorganic content of dry samples was estimated from the residual mass observed at 1000 °C relative to that measured at 150 °C. The organic content of the intercalated bentonites was determined by comparing their mass loss values with that obtained for the neat bentonite. These values were compared with the theoretical organic content calculated on the basis of complete ion exchange equivalent to the CEC (Table 1) [39]. In all cases, the surfactants intercalated to a level that exceeded the CEC theoretical expectation by 10%, 11%, and 21% for the SC14, DC16, and the surfactant mixture intercalated bentonites, respectively [39]. In addition, the DTA results demonstrated that the endothermic peak was below 150 °C for the crude bentonite due to the loss of interlayer water (Figure 5b). The weight loss attributed to the dehydration event below 150 °C corresponded to 17% for the raw bentonite and less than 3% for the modified bentonite. The high amount of water exhibited by the raw bentonite was expected as the Ca^2+^ and Na^+^ ions probably have a higher tendency for hydration than the oleophilic surfactant. The same behavior was observed by Ramos Filho et al. [41].

## 4. Preparation of Biodegradable Polymer/Bentonite Nanocomposites

**Figure 6:**
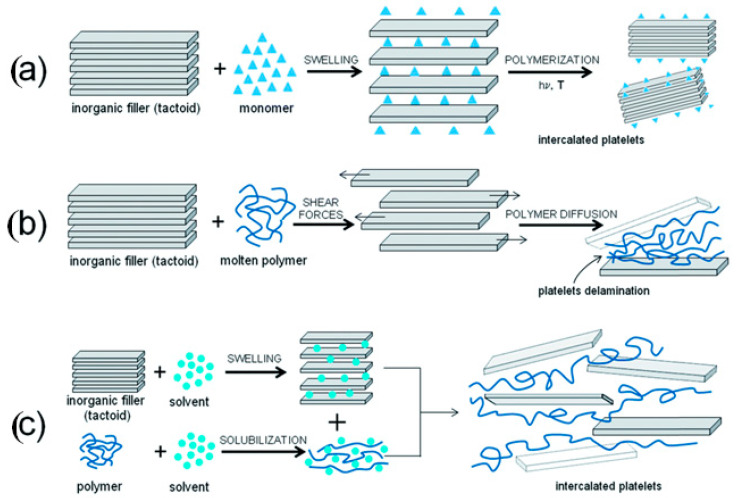
Schematic illustration of (**a**) in situ polymerization, (**b**) melt processing, and (**c**) solution casting methods for the preparation of polymer-based bentonite nanocomposite. Reprinted with permission from Ref. [42]. Copyright 2014, Royal Society of Chemistry.

Generally, three main methods, melt processing, solution casting, and in situ polymerization, are mostly used to prepare polymer-based clay nanocomposites (Figure 6) [42]. Although among these methods solution- and melt-mixing are considered more accessible methods, these may result in less effective polymer intercalation and/or exfoliation of organoclay layers into the polymer matrix due to solvent co-intercalation or for melt processing, slow polymer transport into the interlayer space. Furthermore, they require an additional processing step after polymer synthesis, increasing the cost of the final product [3]. The definition and procedures of the methods of polymer nanocomposites are described below.

*(a) In situ polymerization*. In situ polymerization refers to a process in which the formation of polymers occurs directly at the place in which they will be used, without the need for prior production and further processing of the polymers [43]. In this process, the monomers are introduced at the application site and then the chemical polymerization reactions are initiated, which leads to the formation of the solid polymer. Generally, this polymerization is catalyzed by initiating agents or other reactive agents such as heat, light, or chemical reagents [44]. When this method is used in the preparation of nanocomposites of clays and polymers, nanoclays (typically organically modified ones) and an initiator or catalyst are first dispersed in monomers or a monomer solution, and then polycondensation is performed using standard methods. This method has been frequently used to obtain nanocomposites with a homogeneous distribution of well-exfoliated clay layers in the polymer matrix [45].

The in-situ polymerization method can be used to fabricate product based on polymer nanocomposites to be applied in several applications such as coatings [46], adhesives [47], sealants [48], etc. The method’s main advantage is greater control over reaction conditions, such as temperature, concentration, and reaction time, which can lead to more consistent and controlled properties in the polymers produced [49].

Studies on the development of novel biodegradable polymer-based bentonite nanocomposites prepared via in situ polymerization have been reported [3,50,51]. Salt-resistant poly(acrylic acid-N, N-diethyl acrylamide) [P(AA-DEA)] was designed recently to intercalate and graft calcium bentonites (CaB) via in situ polymerization without prior Na activation [52]. Hydrophilicity and barrier property tests showed that the swell index of CaB/P(AA-DEA)-8% in aggressive CCP leachate (*I* = 176 mM) was 3.4 times that of pristine CaB, giving CaB/P(AA-DEA)-8% a very low *k_CCP_* (1.7 × 10^−12^ m/s), which is only 3.3% of the U.S. standard requirement for geosynthetic clay liners and ~four orders of magnitude lower than the *k* of pristine CaB.

In situ polymerization allows for the formation of polymers directly at the application site, providing advantages such as personalization, efficient adhesion, reduction in intermediate processes, and process control. This technique is widely applied, enabling the development of polymeric and bentonite materials with specific properties to meet the demands of different applications [53]. However, the disadvantages of in situ intercalative polymerization are the slow rate of reaction, and dependence of clay exfoliation through the following processes: swelling of the clay, diffusion rate of monomers in the clay layer gallery, and the fact that oligomer may be formed upon incomplete polymerization [54].

*(b) Melt processing*. Melt processing has been widely used to produce nanocomposites due to numerous advantages, for example, design versatility. The process involves mixing particles with the polymer, in which the mixture is heated to a temperature above the softening point of the polymer. The polymer melt processing is a viable and widely used method in the large-scale production of geometrically complex objects [55]. Melt processing is considered an economical product manufacturing technique. Also, it does not require organic solvents, thus it can be considered an environmentally friendly process. Meting processing allows for compatibility with several industrial methods (e.g., extrusion), facilitating possible scale-up. Moreover, melting processing enables the production of solid and durable joints between materials, ensuring the structural integrity of products when appropriately produced [56].

Poly-lactic acid (PLA)-based bentonite (BNT) composites has been reported [57]. The findings demonstrated the enhancement of mechanical properties (tensile strength, Young’s modulus, and elongation at break). The lowest bentonite content studied for BNT composites (0.5 wt.-%) resulted in superior tensile strength compared to neat PLA. The XRD results from the study showed that the bentonite was well dispersed and exfoliated in PLA. This suggests that the compounding process using extrusion was effective in the preparation of PLA-based bentonite clay nanocomposites.

Melting processing of materials has several advantages in creating finished products or complex components of composites of polymers with bentonite, which can consequently present improved characteristics, such as increased thermal and mechanical resistance [56]. However, there may be restrictions on the size and scale of composites that can be produced. This can be a hindrance to making more extensive or large-scale materials. Therefore, systematically assessing whether fusion processing is suitable for creating the required composite should be crucial. If so, the efficiency and practicality of making the object can be made the most of.

*(c) Solution casting*. Solution casting is widely used in the fabrication of multifunctional composites, such as biomedical materials and adsorbents. Solution casting can be carried out by dissolving a polymer in a suitable solvent. At the same time, other materials are dispersed in the same or different solvent before the dispersions are mixed to generate a homogeneous mixture [58]. The efficient exfoliation of the stacked clay layers using an appropriate solvent has been considered an attractive advantage for the area. The successive addition of a polymer solution to the dispersion of completely delaminated nanoparticles leads to a strong interaction between the polymer macromolecules and individual clay layers. The driving force for the intercalation of the biopolymer into the clay galleries in the solution is the entropy obtained with the desorption of the solvent molecules, which compensates for the entropy decrease in the confined intercalated chains. When the solvent is evaporated, the intercalated structure remains, which results in the final nanocomposites. The disadvantages of this process are mostly due to the solvents used, as most are considered unsafe. In addition, the production of materials using the solution casting method requires attention during the solvent removal step. If the solvent remains in the product, it may reduce the interfacial performance between the polymer and filler [58,59].

A series of novel chitosan–bentonite nanocomposite films were prepared by using solvent casting for diverse applications [60,61,62,63]. Incorporation of an appropriate amount of bentonite into the solution mix relative to chitosan to make the films enabled the improvement of almost all the properties needed (e.g., resistance to folding and the ability to absorb water). Therefore, the solution casting method can be easily applied to produce bentonite and polymer-based composites [60,61,62,63]. An exciting advantage of the process is the possibility of using biopolymers to manufacture composites. Continuous research for the feasibility of solvents that do not harm the environment must be carried out to make the method even more interesting in terms of sustainability [60,61,62,63].

Finally, the adequate choice of the best process to produce composites based on polymer and bentonite must be made based on several aspects, including the characteristics of the polymer, form of bentonite, desired properties of the composite, and processing conditions when choosing the manufacturing method. Furthermore, coupling agents may be necessary to improve the dispersion and interaction between the polymer and bentonite, ensuring a better-quality composite. Therefore, experimentally evaluating different methods and optimizing processing conditions is essential to obtain polymer and bentonite composites with desired properties and adequate performance for the intended application.

### 4.1. Properties of Biodegradable Polymer-Based Bentonite Clay Composites

**Figure 7:**
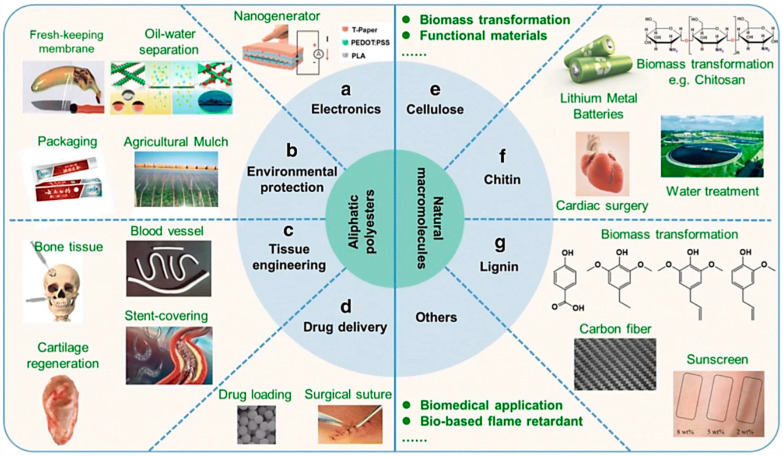
Demonstration of the application of biodegradable polymers in different fields. Reprinted with permission from Ref. [65]. Copyright 2022, Springer Nature.

The better properties of polymer/bentonite nanocomposites compared to the pure polymer used for multifunctional applications such as water treatment and food packaging enhanced properties of polymer/bentonite nanocomposites are achieved when adequate dispersion of nanofillers into the polymer matrix is established. Organoclays such as bentonite can substantially improve various properties of polymers to which they are added. Bentonite can impart high tensile strength and modulus, heat resistance, decreased gas permeability, and flammability and enhanced biodegradability to biodegradable polymers [8,64]. The better properties are obtained due to the strong interfacial interaction between the nanofillers and polymer. Figure 7 demonstrates several applications of biodegradable polymers [65].

### 4.2. Microstructural Properties of Biodegradable Polymer-Based Bentonite Clay Materials

**Figure 8:**
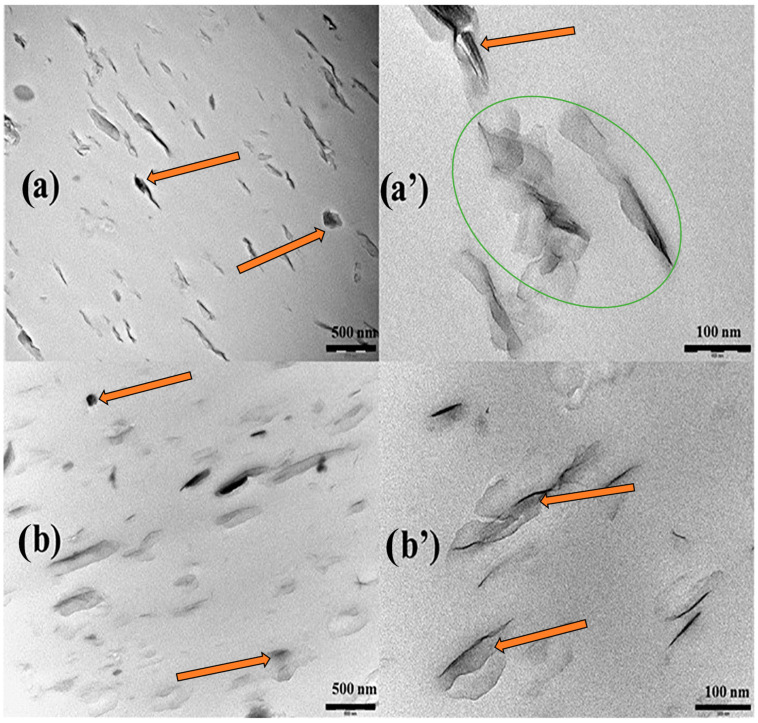
TEM micrographs for PLA-based bentonite nanocomposites. Low (**left**) and high (**right**) magnification TEM micrographs: (**a**,**a′**) PN2HMPEA, (**b**,**b′**) PN2HMPETA. Adapted with permission from Ref. [64]. Copyright 2019, Elsevier.

Experimental measurements and analysis of the microstructure of nanocomposites have been investigated using diffraction techniques, optical and electron microscopy. These methods include scanning electron microscopy (SEM) [66], transmission electron microscopy (TEM) [44], and atomic force microscopy (AFM) [66]. These methods are used to investigate several key factors: filler size and size distribution, the dispersion state of the filler in polymer matrix, and the interfacial adhesion state. In this regard, microstructural studies of polymer–clay bentonite have been investigated. For example, Alves et al. [64] investigated the effect of Brazilian bentonite on the morphological property of PLA nanocomposites using (TEM). The TEM images in Figure 8 demonstrated the dispersion of bentonite clay in the PLA matrix. Small agglomeration of clays in these nanocomposites with numerous individual silicate layers, as well as thin primary clay tactoids are shown by arrows and an oval in Figure 8, and the characteristics of a partially exfoliated nanocomposite structure were obtained. It was demonstrated that uniform distribution of the clay layer into the polymer matrix was achieved. The authors explained that the well-dispersed bentonite clay with thein PLA matrix was a direct consequence of the high intercalation of PLA molecules into the interlayer space of the clay due to the excellent compatibility between the two phases associated with the presence of hydroxyl groups in ethoxylated tallow amine surfactant [64].

**Figure 9:**
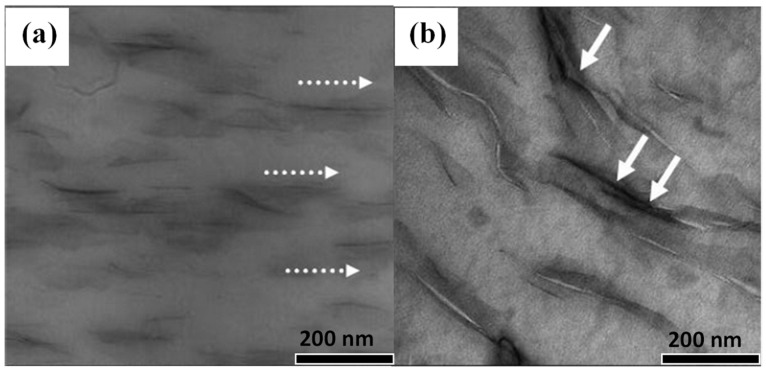
TEM micrographs of PLA-based bentonite nanocomposites compounded at different temperatures: (**a**) 180 °C and (**b**) 220 °C. All samples had 3 wt.-% bentonites added. Reprinted with permission from Ref. [67]. Copyright 2008, Wiley.

Furthermore, a study by Solarski et al. [67] about the effect of processing temperature on the morphology of PLA-based bentonite nanocomposites was examined via TEM. TEM images demonstrated an excellent dispersion of organo-modified bentonite in the PLA matrix with a high extent of exfoliated silicate layers and intercalated structures at a lower mixing temperature of 180 °C (Figure 9a). This was attributed to a higher level of shear stress transfer between the polymer matrix and clay. Furthermore, the TEM micrograph in Figure 9b suggests a reduction in the quality of the orientation of the sheet nanostructures with the direction of extrusion at a high temperature of melt-compounding (220 °C). Therefore, the stack sizes also look larger than the samples prepared at 180 °C. On the other hand, the white arrows in Figure 9a show that the bentonite clay particles are evenly dispersed through the PLA matrix and the orientation of sheet nanostructures in the direction of extrusion are more pronounced. The white arrows in Figure 9b demonstrate that the bentonite clay agglomerates tended to envelope PLA matrix.

**Figure 10:**
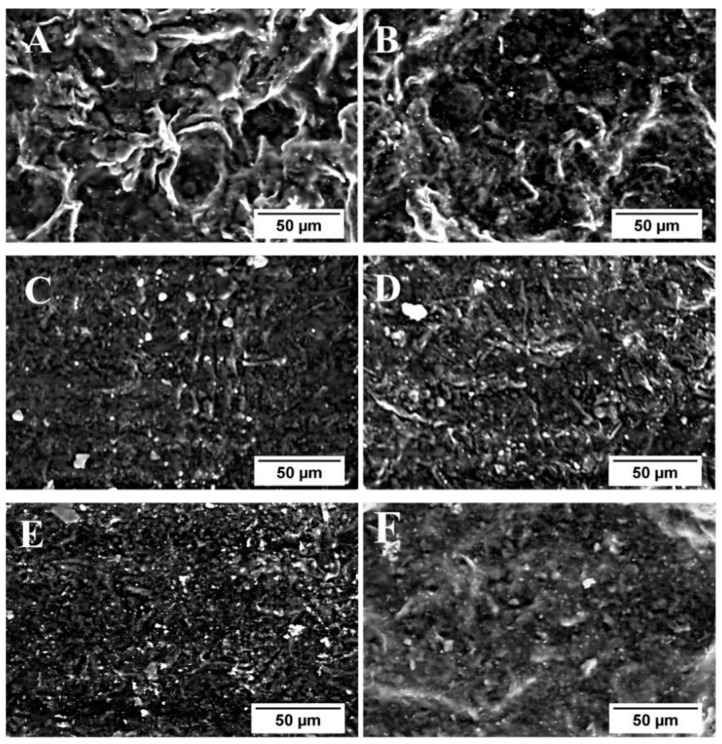
SEM micrographs of the bioplastic films (**A**) without bentonite, (**B**) 0.5 wt.-% bentonite, (**C**) 1 wt.-% bentonite, (**D**) 1.5 wt.-% bentonite, (**E**) 2 wt.-% bentonite, and (**F**) 2.5 wt.-% bentonite. Reprinted with permission from Ref. [68]. Copyright 2021, Elsevier.

Shanmathy et al. [68] demonstrated that bentonite and starch were fully homogenized as they revealed no phase separation, holes, or cracks. The reduced roughness of the films produced in this study was due to the interaction between the negatively charged bentonite and positively charged starch [68]. Figure 10 shows SEM micrographs of the bentonite dispersed in the starch. Different concentrations of bentonite were used. According to the authors, a high concentration of bentonite in the starch reduced the roughness of the biodegradable film. Furthermore, the attraction between the molecules might have helped in reducing the roughness by keeping the contents well intact [68]. In conclusion, the effective dispersibility of bentonite clay into the PLA matrix with incomplete or complete exfoliation structure can be achieved.

### 4.3. Mechanical Properties of Biodegradable Polymer-Based Bentonite Clay Materials

**Figure 11:**
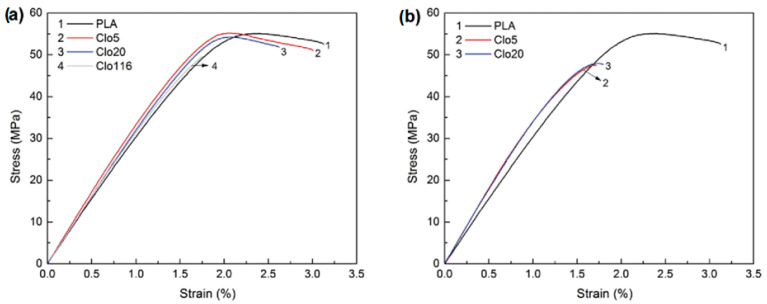
Tensile stress–strain curves of neat PLA and PLA/clay nanocomposites with (**a**) 1 wt.-% and (**b**) 5 wt.-% of bentonite. Reprinted with permission from Ref. [72]. Copyright 2018, Elsevier.

In general, the most often tested mechanical properties in polymer nanocomposites include Young’s modulus, tensile strength, and elongation at break. These mechanical properties are influenced by several factors such as polymer structure, filler content and type, processing methods, and the presence of additives (such as plasticizers) [69]. Furthermore, the interfacial interactions between the biodegradable polymer and the nanofiller (bentonite clay) have a noteworthy effect on the mechanical properties of biodegradable polymer composites [70,71]. For example, Paspali et al. [72] investigated the effect of three bentonites (Clo5, Clo20, and Clo116) on the mechanical properties of PLA composites. Neat PLA and PLA–bentonite nanocomposite samples demonstrated an initial linear elastic deformation, followed by non-linear deformation (Figure 11). Figure 11 shows that the clay content and type substantially influenced the mechanical behavior of the PLA-based bentonite nanocomposites compared to the neat PLA matrix. Another study by Ollier et al. [8] about the effect of organo-modified bentonite on the mechanical properties of the polycaprolactone (PCL) matrix showed that PCL-based bentonite clay nanocomposites had improved tensile mechanical properties such as Young’s modulus and tensile strength compared to the neat PCL matrix (Table 2). This behavior was attributed to the excellent dispersion of the bentonite nanofiller into the polymer. In addition, other studies also reported that the degree of dispersion of organoclay plays an important role in enhancing the viscoelastic behavior of the polymeric matrix [73,74]. Petersson and Oksman [75] also evaluated the mechanical properties of PLA/bentonite nanocomposites. In their results, the authors demonstrated a 53% improvement in tensile modulus and a 47% increase in yield strength compared to pure PLA (Table 2).

In the study by Behera et al. [76], an increase in bentonite clay concentration was observed to improve the mechanical properties of bioplastic films. This might have been due to the homogeneous dispersion of bentonite in the bioplastic composite film. Table 2 thus shows that with the increase in the concentration of bentonite, the tensile strength and Young’s modulus increased [8,75,76].

### 4.4. Thermal Stability of Biodegradable Polymer/Bentonite Clay Nanocomposites

The thermal stability of polymeric materials is typically investigated via thermogravimetric analysis (TGA). Generally, the addition of inorganic nanofillers has been reported to enhance the thermal stability of biodegradable polymers [78,79]. Ray and Bousmina [80] reported that the silicate layers act as a barrier for incoming gases and gaseous by-products formed during degradation, improving the thermal stability of the polymeric materials. The extent of this increase usually depends on the degree of exfoliation of the organoclays [81,82,83].

**Figure 12:**
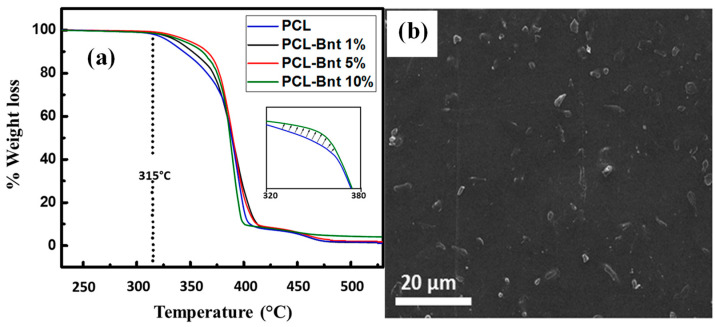
(**a**) TGA profiles of the virgin PCL and PLC-based organo-modified bentonite nanocomposites (1, 5, and 10 wt.-% of nanofiller) and (**b**) SEM micrograph of the PCL–organo-modified bentonite nanocomposites (5 wt.-% of clay). The subfigure inserted in (**a**) shows that at 320 °C to 380 °C, the PCL-Bnt 10% composite demonstrated to be more stable than neat PCL. Reprinted with permission from Ref. [51]. Copyright 2021, Elsevier.

Studies on the thermal stability of the biodegradable polymer/bentonite nanocomposites play an essential function in deciding their working temperature limit and the environmental conditions for use, which are related to their thermal decomposition temperature and decomposition rate [82,84]. For example, Nabgui et al. [51] used TGA to investigate the effect of organo-modified bentonite (OBnt) on the poly(ε-caprolactone) (PCL) matrix. Figure 12a shows the TGA thermograms of the virgin PCL and the obtained poly(ε-caprolactone) based organo-modified bentonite nanocomposites with different concentrations of nanofiller. The mass loss of the virgin PCL, representing thermal degradation, starts at 315 °C. In contrast, the mass loss of the PCL–bentonite bionanocomposites, which corresponds to thermal degradation, occurs at a slightly higher temperature (320 °C). At the second degradation stage (up to 330 °C), the shift to a higher thermal degradation temperature was more significant for the bio-composite material (PCL–OBnt) compared to the virgin PCL. The results demonstrated that the presence of clay fillers such as organo-modified bentonite results in thermal stability improvement. The similar trend of mass loss behavior of the PCL-based bentonites (1 wt.-% and 5 wt.-%) composites could be due to the formation of reasonably large amounts of cross-linked carbonaceous species for PCL-based bentonite composites (5 wt.-%) which undergo slow decomposition within a certain temperature range, particularly at high temperatures [85,86,87]. Moreover, good dispersion of the bentonite clay nanofiller in the polymeric matrix (Figure 12b) could have enhanced the nanocomposite stability through the formation of protecting layers [88,89,90,91,92,93]. Furthermore, the absence of holes and phase separation confirm the significant interaction between PCL and organo-modified bentonite [51]. Additionally, Table 3 summarizes previous works on bentonite as a filler for biodegradable polymer nanocomposites.

### 4.5. Difficulties in Clay/Biodegradable Polymer Nanocomposite Development

The production of clay/polymer composites faces several challenges that can make obtaining materials with desired properties difficult. The adequate clay dispersion of clay to form intercalated or exfoliated structures within the polymer is a challenge in producing polymer composites. Clay is composed of nanometer-sized particles that tend to agglomerate, forming aggregates [94]. To obtain high-quality composites, obtaining a uniform dispersion of the clay particles in the polymeric matrix is essential. There needs to be a better understanding of the mechanisms related to getting polymer composites with superior properties due to the lack of knowledge about the polymeric behavior in each system.

Another challenge is the thermal stability of organic clays, which have attracted the interest of many researchers. Organic clays modified with ammonium salts are widely used to prepare clay and polymer composites. However, many of these ammonium salts are thermally unstable and can degrade during processing or during the course of using the composite. This can result in a loss of composite properties and stability over time. Also, the long-term stability of biodegradable polymer–clay nanocomposites based on modified organoclays is a concern [95].

Finally, the large-scale production of clay/polymer composites is another challenge. Consistent reproduction of characteristics and properties on an industrial scale can be difficult due to factors such as the variability of raw materials, processing control, and optimization of manufacturing conditions. These challenges highlight the importance of careful approaches and formulation strategies to achieve proper dispersion, polymer–clay compatibility, and management of composite properties. Processing techniques and choosing suitable additives are also vital for overcoming challenges and obtaining clay/polymer composites with improved performance.

### 4.6. Designing Biodegradable Polymer/Clay Nanocomposites: Theories and Models

Theory and modeling are essential tools for designing biodegradable polymer/clay nanocomposites with desired properties. These approaches help understand the interactions between the polymer and the clay, predict the behavior of the composite, and optimize its formulation. Modeling approaches employed for polymeric nanocomposite systems generally consist of three categories of scaling methods: (i) molecular, (ii) microscale, and (iii) meso/macro scale, according to different size effects [96]. Molecular-scale methods focus on molecular dynamics, the Monte Carlo molecular method, and the molecular mechanics of atoms, molecules, or groups of units. On the other hand, microscale methods tend to bridge the gap between molecular-scale methods and meso/macroscale methods [97].

Various methods, such as Brownian dynamics and the Boltzmann lattice method, have been developed to study microscopic structures and the exchange of composite constituents [96]. Therefore, some considerations must be made in this approach relating theory and modeling, for example, the theory of mixing, interaction, and dispersion mechanisms.

The mixture theory explains the interaction between polymeric phases and clay particles. Theoretical models, such as the Halpin–Tsai model, Mori–Tanaka model, and Hirsch model are applied to estimate the mechanical properties of the composite based on the properties of individual phases and morphology of the composite [96,98].

The Hirsch model (Equation (1)) was developed to estimate the modulus of the elasticity of composites with a combination of parallel and series models, in which the equation is the effect of the matrix modulus, the load modulus, and the volume fractions together with an empirical constant [99]. The Hirsch model equation is given as [99]:

(1) $$E_{c}=\chi \left(E_{m}\left(1-\phi_{p}\right)+E_{p}\phi_{p}\right)+(1-\chi )\frac{E_{p}E_{m}}{E_{m}\phi_{p}+E_{p}(1-\phi_{p})}$$

where χ is an empirical constant that controls the stress transfer between the fillers and matrices in composites, which is obtained through the curve-fitting of experimental data (0 < χ < 1); $E_{c}$, $E_{m}$, and $E_{p}$ are the moduli of the composite, matrix, and filler, respectively; and $\phi_{p}$ is the volume fraction of filler in composite.

The Halpin–Tsai model provides a prediction for the elastic moduli (Equation (2)) of unidirectional composites with respect to volume fraction and load geometry, where the model is generally used for continuous or discontinuous fillings [100]. The Halpin–Tsai equations are based on the “self-consistent micromechanics method” developed by Hill. Hermans employed this model to obtain a solution in terms of Hill’s “reduced moduli”. Halpin and Tsai reduced Hermans’ solution to a simpler analytical form and extended its use to a variety of filament geometries. The Halpin–Tsai model equation is given by [100]:

(2) $$E_{c}=E_{m}\frac{1+\xi \eta_{L}\phi_{p}}{1-\eta_{L}\phi_{p}}$$

where $\eta_{L}$ and ξ (constants that depend on the geometry and aspect ratios of fillers in the composite) are given by Equations (3)–(5) [86]:

(3) $$\eta_{L}=\frac{\left(\frac{E_{p}}{E_{m}}\right)-1}{\left(\frac{E_{p}}{E_{m}}\right)+\xi }$$

(4) $$\xi =2\alpha =2\left(\frac{l}{t}\right)\;for\;longitudinal\;modulus\;(E_{11})$$

(5) $$\xi =2\;for\;tranverse\;modulus\;\left(E_{22}\right)$$

where l and t are the length and thickness/depth of dispersed fillers in the composite, respectively.

Regarding dispersion, excellent clay dispersion in the polymer is crucial for obtaining improved properties, whereas modeling can help predict the agglomeration of the clay particles and optimize the processing parameters to achieve uniform distribution. Computer simulations, such as molecular dynamics and computational fluid mechanics, can be used to investigate the dispersion and interaction of clay particles within the polymeric matrix [97]. Finally, modeling can help understand the interaction mechanisms between the polymer and clay. This involves the analysis of intermolecular forces, chemical bonds, and surface interactions between the clay particles and polymer chains [98]. Danusso and Tieghi presented a relationship (rigid matrix-based composites) of mechanical strength and volume fraction Equation (6) [101]:

(6) $$\sigma_{c}=\sigma_{m}(1-\psi )$$

where $\sigma_{c}\;$ and $\sigma_{m}$ are the tensile strengths of the composite and matrix, respectively, and ψ is the area fraction in the cross section.

Another model was proposed by Nicolais and Narkis [102]. The model is based on the Danusso and Tieghi model and was obtained by replacing the volume fraction with a power law function in terms of volume fraction Equation (7) [102]:

(7) $$\sigma_{c}=\sigma_{m}(1-a\phi_{p}^{b})$$

where a and b are constants influenced by particle shape and arrangement in composites.

Theoretical models can be developed to describe these interactions and predict the resulting properties [103]. A theoretical modeling framework was reported for predicting the tensile modulus and tensile strength of biodegradable bioepoxy/clay nanocomposites in terms of clay and epoxidized soybean oil (ESO) content [96]. Random orientation of dispersed clay fillers was found to be a significant factor in predicting the elastic modulus of nanocomposites at clay contents of 1–8% by weight (ESO content: 20% by weight) according to the Hui–Shia laminated model (HS) and Halpin–Tsai laminated model (HT). Furthermore, when the clay content was set at 5% by weight, the HS laminate model corresponded well with the experimental data of bioepoxy/clay nanocomposites at ESO contents of 0–40% by weight. In contrast, the Hirsch model predicted values closer to the experimental data at the ESO content of 60% by weight.

Therefore, modeling and simulation are powerful tools for producing composites based on polymers and clays, which must be experimentally validated to ensure their accuracy and reliability. Combining theoretical and experimental approaches allows for a more comprehensive understanding of polymer/clay nanocomposites. It helps direct the design and development of materials with required properties and reduce the environmental impact.

## 5. Conclusions

This review explores the thermal, morphological, and mechanical properties of bentonite clay composites based on biodegradable polymers for multifunctional applications. In addition, we present and the discuss processing methods, theory, and modeling relating the subject. Polymeric/clay composites have aroused great interest due to the combination of the clay’s unique properties with the polymer’s properties, resulting in materials with improved performance and greater versatility. The incorporation of bentonite clay in biodegradable polymers can lead to an increase in the thermal stability of the composites. This is due to clay’s ability to act as a thermal barrier, thereby slowing polymer degradation during heating. In addition, bentonite clay can promote higher crystallinity in polymer composites, resulting in better thermal resistance.

Regarding morphological properties, microscopic analysis in several studies revealed that a uniform dispersion of clay in the polymeric matrix could provide composites suitable for several applications. Furthermore, incorporating bentonite can significantly increase the tensile strength of polymer-based composites. Rigidity and impact strength have been attributed to the incorporation of clay in the polymer matrix, a mechanical reinforcement in the polymeric matrix.

There are several clay/polymer processing methods: melt processing, solution casting, in situ polymerization, etc. An appropriate choice of the processing method is required according to the desired properties of the composite. Finally, modeling and simulation have been powerful tools in producing composites based on polymers and clays, in which the union between experimental and numerical studies has ensured reliability in research in the field. In summary, these materials represent a sustainable and viable alternative to replace conventional non-biodegradable plastic products, contributing to the preservation of the environment. In addition, continuous research and advances in the area have enabled the exploration of the full potential of these composites, thereby driving the development of innovative solutions for various applications.

## Figures and Tables

**Table 1:** Estimated organic content from the TG results.

Sample	Residual Mass (%), 150 °C	Residual Mass (%), 1000 °C	Theory Organic Content (%)	Experimental Organic Content (%)
Crude bentonite	83.4	79.3	-	-
SC14	97.1	71.9	19.0	20.9
SC14 + DC16	97.9	60.5	28.1	34.0
DC16	98.1	55.8	35.3	39.3

**Table 2:** Mechanical properties of biodegradable polymer-based bentonite nanocomposites.

Sample	Young’s Modulus (MPa)	Tensile Strength (MPa)	Reference
Neat PCL	114.8 ± 2.0	12.4 ± 0.2	[8]
PCL–1.5 wt.-% Bent	194.7 ± 12	13.9 ± 0.2	[8]
PCL–3.0 wt.-% Bent	180.8 ± 22.1	13.6 ± 0.5	[8]
PCL–1.5 wt.-% bCBK	203.2 ± 20.9	19.3 ± 0.8	[8]
PCL–3.0 wt.-% bCBK	257.3 ± 37.0	19.8 ± 3.1	[8]
Neat PLA	28.5 ± 3.8	1.7 ± 0.2	[75]
PLA/Bentonite	42.0 ± 4.3	2.6 ± 0.3	[75]
Starch films	40.9 ± 6.04	2.56 ± 0.13	[76]
Starch/Bentonite (0.5 wt.-%)	45.35 ± 3.03	2.66 ± 0.72	[76]
Starch/Bentonite (1 wt.-%)	54.38 ± 7.89	3.053 ± 0.361	[76]
Starch/Bentonite (1.5 wt.-%)	72.65 ± 11.42	4.063 ± 0.12	[76]
Neat PLA	452 ± 66	37.0 ± 6.0	[77]
PCL/Bentonite (5 wt.-%)	767 ± 61	42.3 ± 2.5	[77]

**Table 3:** Summary of bentonite as a filler for biodegradable polymer nanocomposites.

Biodegradable Polymer/Bentonite Clay Nanocomposites	Remarks	Reference
Poly(hydroxybutyrate)/polyethylene glycol blend (PHB/PEG) with organobentonite (1 wt.-% and 3 wt.-%)	It was observed that the initial temperature for the degradation of bionanocomposites showed different behavior for organobentonite (1 wt.-% and 3 wt.-%). The thermal stability of bionanocomposites increased with the organobentonite content. Furthermore, it was also verified that clay addition to most systems led to an increase in crystallinity compared to the PHB matrix, which was attributed to the clay nucleating effect.	[2]
Poly(butylene adipate) (PBA)/organoclay nanocomposites	The organophilic modification was observed to promote efficient clay delamination, resulting in nanocomposites with a predominantly exfoliated morphology when the appropriate organoclay (i.e., with the optimal organomodifier/clay ratio) in the appropriate concentration range of 2–3 wt.-% was used. The increased crystallinity of PBA suggests that the nanoclay acts as a heterophase-nucleating agent. The enhanced thermal stability of the PBA matrix confirmed the good quality of the delamination and dispersion of the clay lamellae in the polymer matrix.	[3]
Polycaprolactone/organobentonite nanocomposites	The morphology characterization of the nanocomposites showed that the organo-modification of the clay greatly improved its dispersion in the polymer matrix. As a consequence, it is demonstrated that the reinforcement of PCL with 3 wt.-% loading of organoclay produces the strongest improvement in creep resistance. The instantaneous creep strain and experimental creep rate decrease by more than 9% and 27%, respectively.	[8]
Polyester resin (Dolplast)/bentonite nanocomposites	The results clearly showed that the chemical modifications of the bentonite clay caused a desired effect on its final properties (thermal, barrier (water absorption), mechanical (flexural), and dynamic mechanical properties), improving the performance of the nanocomposites. The enhancements were directly related to the dispersion of the clay inside the matrix viewed via transmission electron microscopy.	[24]
Poly(ε-caprolactone)/bentonite nanocomposites	The prepared poly(ε-caprolactone)/bentonite presented better thermal stability regardless of the content of bentonite as a reinforcing filler. In fact, the melting point and poly(ε-caprolactone)/bentonite degree of crystallinity determined via TGA and DSC analyses, increased by increasing the amount of the filler. Furthermore, the fractured surfaces of the neat polymer (PCL) and poly(ε-caprolactone)/bentonite were compared using SEM, whereby the adhesion between clay particles and the polymer was clearly exposed, as well as the dispersion of the bentonite filler in the polymeric matrix. Finally, the bio-composites with 5 wt.-% of bentonite in the poly(ε-caprolactone) matrix displayed the highest resistance under stress with 66 MPa instead to 46 MPa only for the neat poly(ε-caprolactone).	[51]
PLA/bentonites nanocomposites	Nanocomposites were prepared at low clay compositions of 0.5, 1, 3, and 5 wt.-% of bentonite. From the XRD spectra, the partial exfoliation of the nanoclay layers occurred during the melting extrusion. This resulted in improvement of mechanical properties, such as Young’s modulus, tensile strength, and elongation at break. The highest tensile strength was obtained with the addition of 0.5 wt.-% commercial bentonite, increasing about 23.25% compared to the neat PLA. The increasing composition of the clays revealed a decrease in mechanical properties due to filler–filler interactions. Furthermore, the water absorption of nanocomposites up to 1 wt.-% of clays was better than that of the neat PLA. Biodegradability was enhanced in the presence of a higher clay composition due to the high hydrophilicity of clay, high water uptake, and high interactions.	[57]
Chitosan/bentonite nanocomposites	The characterization of the chitosan/bentonite composite via FT-IR and XRD indicates evidence of the interaction between the functional groups of chitosan with bentonite, which is beneficial for the adsorption of hexavalent chromium. SEM micrographs of the chitosan/bentonite composite revealed its slightly smooth surface with cotton-like accumulation of irregular shapes. After adsorption, the surface morphology of the chitosan/bentonite composite remained practically unchanged.	[60]
Chitosan/bentonite nanocomposites	The good mechanical structure was confirmed through the tensile strength and elongation values. The chitosan/bentonite composite presented a tensile strength of 40.5 ± 1.6 MPa and elongation of 60.0 ± 0.5%. These mechanical properties were better in relation to the chitosan film (pure chitosan), confirming that the insertion of bentonite was favorable. SEM micrographs demonstrated the presence of bentonite particles in the chitosan/bentonite composite. Additionally, the results showed that the chitosan/bentonite composite presented a heterogeneous structure.	[61]
Chitosan/bentonite nanocomposites	The incorporation of an appropriate amount of bentonite in the solution mixture with respect to chitosan in order to fabricate nanocomposite films dramatically improved almost all the properties required for an ideal wound dressing material. FTIR spectra confirmed the H-bonding interactions between the hydroxyl group of bentonites with the hydroxyl and amino groups of chitosan. Further, the SEM images showed a rough surface of the nanocomposite film that might be due to the entrapment of undissolved bentonite particles in the nanocomposite film.	[63]
PLA/organoclay nanocomposites	The results showed that the hybrid organo-modified montmorillonites with di(alkyl ester) dimethyl ammonium chloride (EA) and ethoxylated tallow amine (ETA) (both containing polar groups) conferred compatibility between the organoclays and PLA, while the phosphonium compound unexpectedly failed to promote the dispersion of the clay layers in PLA nanocomposites, related to its poor compatibility with PLA. However, these systems may be useful when combined with other polymers. In addition, the use of hybrid organo-modified montmorillonites (OMt) may be a good approach to obtain polymer clay nanocomposites with final desirable properties, depending on the type, amount, and surfactant combinations in the hybrid system, being a field that still has to further be explored. DSC showed dependence on the nanocomposite thermal behavior with the dispersion level of the organoclays. In general, the presence of organoclays reduced the glass transition temperature of PLA and its cold crystallization temperature, and increased its crystallinity, which was related to an effective heterogeneous crystal nucleation promoted by the organoclay.	[64]
Polylactide/bentonite nanocomposites	Because of good compatibility with the PLA matrix, the dispersion of bentonite (B104) occurred under different conditions without difficulty. The results obtained showed that at low temperatures of mixing, the shear stress exerted on the polymer had a key role in the extent of intercalation and delamination. Bentonite could be added up to 4 wt.-% into PLA without detrimentally sacrificing the tensile strength of melt-spun filaments, especially at a high draw ratio. Interestingly, the composites-based multifilament was knitted, and the flammability studied using cone calorimeter at 35 kW/m^2^. A strong decrease, up to 46%, in the heat release rate was observed.	[67]
Taro starch/bentonite nanocomposites	SEM and FTIR results showed that the films formed were homogeneous and confirmed the functional groups of Taro starch. With the increase in bentonite concentration, the tensile strength of the bioplastic film was found to increase. The bentonite films exhibited more resistance to salt and acid, were susceptible to alkali, and showed minor swelling.	[68]

## Data Availability

Not applicable.

## Abbreviations

ABS	Acrylonitrile–butadiene–styrene
BNT	Bentonite
[P(AA-DEA)]	Poly(acrylic acid-N, N-diethyl acrylamide)
CaB	Calcium bentonites
CCP	Coal combustion product
*k_CCP_*	Hydraulic conductivity
ESO	Epoxidized soybean oil
MMT	Montmorillonite
MB	Modified bentonite
OMMT	Organo-montmorillonite
OBnt	Organo-modified bentonite
HS	Hui–Shia laminated model
HT	Halpin–Tsai laminated model
χ	Empirical constant to control the stress transfer between the fillers and matrices
*E_c_*	Modulus of the composites
*E_m_*	Modulus of the matrices
*E_p_*	Modulus of the fillers
*ϕ_p_*	Volume fraction of the fillers in the composites
*η_L_*	Stress-partitioning factor
*ξ*	Constant depending on the geometry and aspect ratios of the fillers in the composites
*l*	Length of dispersed fillers in the composites
*t*	Thickness/depth of dispersed fillers in the composites
*σ_c_*	Tensile strength of the composites
*σ_m_*	Tensile strength of the matrices
*ψ*	Area fraction in the cross section
*a*	Constant influenced by the particle shape and arrangement in the composites
*b*	Constant influenced by the particle shape and arrangement in the composites
